# Additional burden of asymptomatic and sub-patent malaria infections during low transmission season in forested tribal villages in Chhattisgarh, India

**DOI:** 10.1186/s12936-017-1968-8

**Published:** 2017-08-08

**Authors:** Mehul Kumar Chourasia, Kamaraju Raghavendra, Rajendra M. Bhatt, Dipak Kumar Swain, Hemraj M. Meshram, Jayant K. Meshram, Shrity Suman, Vinita Dubey, Gyanendra Singh, Kona Madhavinadha Prasad, Immo Kleinschmidt

**Affiliations:** 1 National Institute of Malaria Research (ICMR) IIR-WHO Project, Field Unit, Kondagaon, Chhattisgarh India; 2 National Institute of Malaria Research (ICMR), Sector-8, Dwarka, New Delhi 110077 India; 3 National Institute of Malaria Research (ICMR), Field Unit, Lalpur, Raipur Chhattisgarh India; 4 Department of Infectious Disease Epidemiology, London School of Hygiene and Tropical Medicine, London, UK

**Keywords:** Malaria, Asymptomatic, Sub-patent, PCR, Chhattisgarh, India

## Abstract

**Background:**

The burden of sub-patent malaria is difficult to recognize in low endemic areas due to limitation of diagnostic tools, and techniques. Polymerase chain reaction (PCR), a molecular based technique, is one of the key methods for detection of low parasite density infections. The study objective was to assess the additional burden of asymptomatic and sub-patent malaria infection among tribal populations inhabiting three endemic villages in Keshkal sub-district, Chhattisgarh, India. A cross-sectional survey was conducted in March–June 2016, during the low transmission season, to measure and compare prevalence of malaria infection using three diagnostics: rapid diagnostic test, microscopy and nested-PCR.

**Results:**

Out of 437 individuals enrolled in the study, 103 (23.6%) were malaria positive by PCR and/or microscopy of whom 89.3% were *Plasmodium falciparum* cases, 77.7% were afebrile and 35.9% had sub-patent infections.

**Conclusions:**

A substantial number of asymptomatic and sub-patent malaria infections were identified in the survey. Hence, strategies for identifying and reducing the hidden burden of asymptomatic and sub-patent infections should focus on forest rural tribal areas using more sensitive molecular diagnostic methods to curtail malaria transmission.

## Background

The intensification of intervention measures, such as long-lasting insecticidal nets (LLINs), indoor residual spraying (IRS) of insecticides, artemisinin-based combination therapy (ACT) and improved surveillance methods, have resulted in a 37% reduction in malaria incidence globally and a 60% reduction in the malaria mortality rate between 2000 and 2015. Globally a target of a 90% reduction in malaria incidence and mortality has been set for 2030 [1, 2].

India reported a more than 50% reduction in malaria cases and mortality from 2001 to 2015 [3]. In the year 2016, India launched its strategic plan, the “National Framework for Malaria Elimination in India (2016–2030)”, which focuses on strengthening surveillance by use of bivalent rapid diagnostic tests (RDT), case management with ACT and distribution of LLINs including capacity building, and resource mobilization [4].

India has a complex geographic structure with variable micro and macro ecotype system and malaria endemicity, where many states are free from malaria, whilst in a few states such as Assam and Tripura in the North East, and the central states (Chhattisgarh, Jharkhand, and Madhya Pradesh) are highly malaria endemic. Chhattisgarh is one of the tribal-populated states (33%) in the country, contributing 25% of total reported malaria cases in India [3, 5].

Despite sustained efforts, the number of malaria cases has not been reduced as anticipated. There could be several causative factors such as an increase in drug resistance, insecticide resistance, or mixed infections etc. [6, 7]. Also several studies from African countries had pointed out the important role of asymptomatic, latent and gametocyte carriage in the maintenance of continuous transmission [8–12]. In areas with low vector density and low transmission, asymptomatic and sub-patent malaria poses a challenge for malaria elimination [13, 14].

Sub-patent malaria is difficult to detect due to the limitations of routine diagnostic tools. Low-density parasite infections can be undetectable and thereby cause continued transmission in malaria endemic areas [13]. Previous studies have clearly suggested that polymerase chain reaction (PCR) detects more than double the number of *Plasmodium falciparum* infections compared to light microscopy (LM) and rapid diagnostic test (RDT) in low transmission settings [15]. Even, in highly endemic forest regions, asymptomatic carriers had a high number of gametocytes in their blood and, therefore, sustain malaria transmission [8, 16]. In such areas, PCR apart from being a confirmatory tool for diagnosis of malaria can be useful to detect low parasite density [17, 18].

The Implication of Insecticide Resistance (IIR) project has been implemented in Keshkal, one of the sub-districts of Kondagaon district in Chhattisgarh to study the impact of insecticide resistance in *Anopheles culicifacies* on vector control interventions LLINs and IRS in controlling malaria transmission [19]. In the study area LLINs were distributed with 90% household coverage. Active fortnightly surveillance and yearly cross sectional survey results had suggested that one primary health centre (PHC) area was contributing nearly 40% of the total malaria burden of Keshkal. The possibility of malaria transmission through asymptomatic and sub-patent infection, which were missed by light microscopy and RDTs, poses additional challenges for malaria control efforts in the area. This study was undertaken to assess the burden of asymptomatic and sub-patent malaria infections and their role in malaria transmission in a sample of the population.

Prevalence of submicroscopic infection is largely unknown in India [20–22]. This study quantified the additional burden of asymptomatic and sub-patent malaria infection, and compared three diagnostic methods in the low transmission season in the villages of Keshkal sub-district, Chhattisgarh, India.

## Methods

### Study area and population

**Fig. 1:**
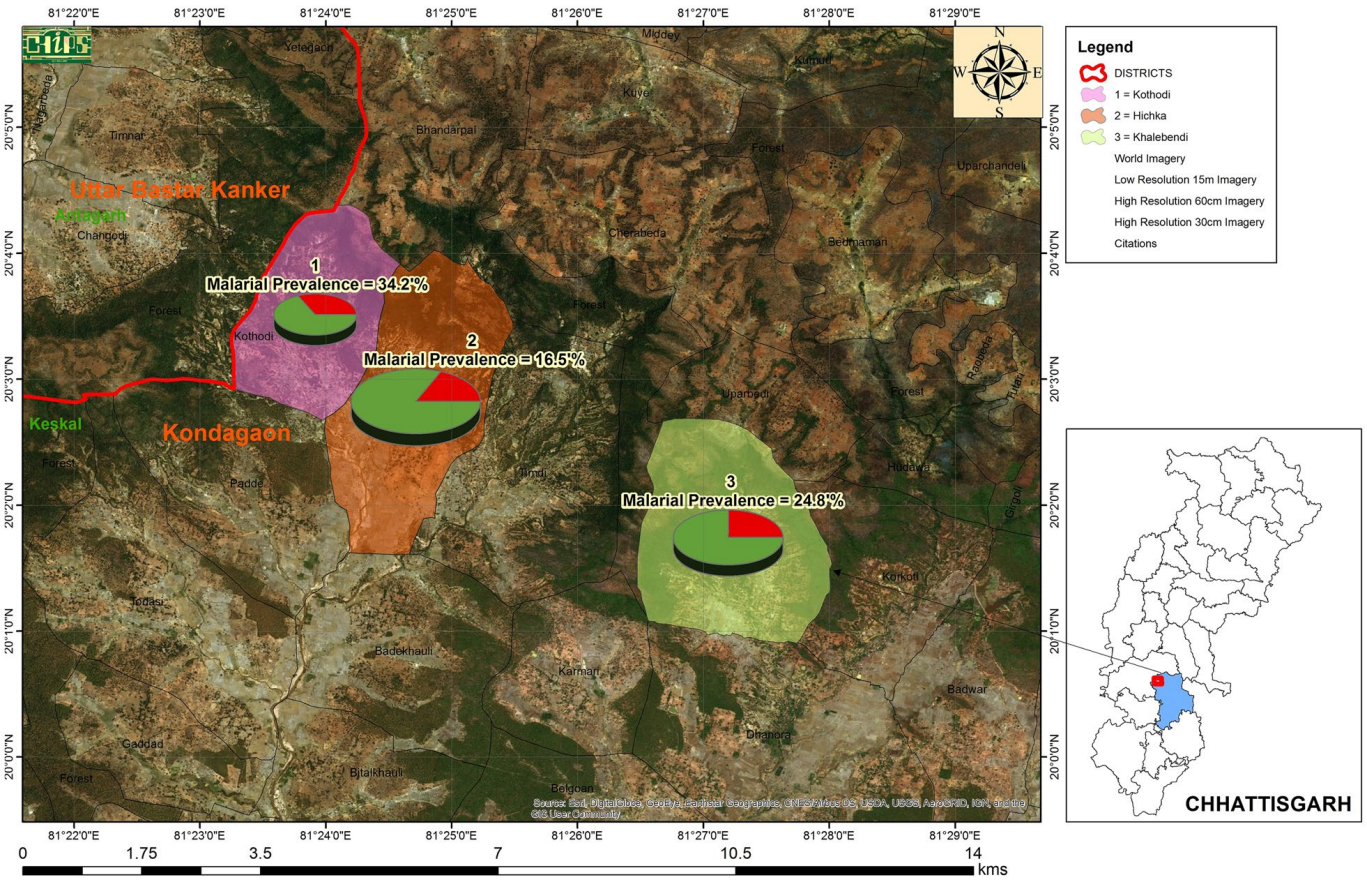
Study sites and village wise spatial distribution of malaria prevalence (with microscopy)

This study was carried out in the villages of Keshkal sub-district, Chhattisgarh, India (Fig. 1) located on banks of a river surrounded by hills and characterized by thick forest mainly inhabited by three tribal communities Gond, Halba and Muria. The study was conducted in the three forested villages (Kothodi, Khalebedi and Hichka) of two sub-centres (Badekholi and Korkoti) under Dhanora Primary Health Centre (PHC) from sub-district Keshkal, with a total population of 885 in 195 households. The livelihood of these communities is primarily based on forest products and agriculture and government supported labor work. Malaria transmission occurs during July to November months every year. *Anopheles culicifacies* is the primary vector in the study area. In the past two decades, IRS using alpha-cypermethrin (synthetic pyrethroid insecticide, at 25 mg/m^2^, twice a year) has been sprayed as the main vector control intervention carried out by the state health department in the study area. The vector is reported resistant to DDT and alpha-cypermethrin in the study area [23].

Data collected from the state health department showed that the annual parasite incidences (API) ranged from 0 to 16 between 2012 and 2015 in the study villages, with higher API in Khalebedi compared to other two villages (Table 1). Since 2015, active fortnightly surveillance for malaria had been conducted in all the study clusters by deploying malaria surveillance workers. During routine surveillance, every month one or two febrile malaria positives were detected in the study area, but in a cross sectional survey conducted in December 2015, the overall positivity rate ranged from 8.6 to 45.6% (Table 2).

**Table 1:** Malaria incidence in study villages by year

Village	Hichka				Kothodi				Khalebedi			
Year	2012	2013	2014	2015	2012	2013	2014	2015	2012	2013	2014	2015
Population	399	418	404	399	183	184	186	183	300	300	307	300
BSC	108	104	55	21	33	75	16	0	83	27	274	31
Total positive	4	1	0	0	0	1	0	0	1	1	7	5
API	10.0	2.4	0.0	0.0	0.0	5.4	0.0	0.0	3.3	3.3	22.8	16.7
SPR	3.7	0.96	0.0	0.0	0.0	1.3	0.0	0.0	1.2	3.7	2.5	16.1

*Source* Community Health Centre, Keshkal

*BSC* blood slide collection, *API* annual parasite incidence, *SPR* slide positivity rate

**Table 2:** Malaria incidence by active fortnightly surveillance of fever cases in the study villages

Village	Hichka			Kothodi			Khalebedi			Method of detection
Month	BSC	TP	SPR	BSC	TP	SPR	BSC	TP	SPR	
May-15	1	0	0	2	2	100	2	0	0	Fortnight surveillance
June-15	1	1	100	1	1	100	0	0	0	Fortnight surveillance
July-15	2	2	100	0	0	0	4	2	50	Fortnight surveillance
August-15	3	0	0	2	1	50	7	1	14.3	Fortnight surveillance
September-15	4	0	0	0	0	0	7	1	14.3	Fortnight surveillance
October-15	4	0	0	3	0	0	2	0	0	Fortnight surveillance
November-15	3	0	0	6	3	50	3	0	0	Fortnight surveillance
December-15	80	11	13.8	75	34	45.3	80	7	8.6	Cross sectional survey
January-16	5	1	20	2	1	50	4	0	0	Fortnight surveillance
February-16	6	0	0	3	0	0	4	0	0	Fortnight surveillance
March-16	3	0	0	2	1	50	4	0	0	Fortnight surveillance
April-16	5	0	0	4	2	50	9	4	44.4	Fortnight surveillance
Total	117	15	12.8	100	45	45	126	15	11.9	Fortnight surveillance

*API* annual parasite incidence, *SPR* slide positivity rate, *BSC* blood slide collection, *TP* total positive cases

### Demographic data collection

A census of all the households was prepared and all households and residents were enumerated. A community-wide cross-sectional survey for malaria infection was carried out in the dry season (March–June 2016) that included all inhabitants older than 6 months. During the survey, all members who were available at home were invited to participate in the study. Study purpose, rationale and methods of the study were described to the head of the household and their family members. Written informed consent was sought from the head of the household, while verbal consent was obtained from other members of the family for participation in the study. A primary exclusion criterion was participants should neither have had malaria in the past two weeks preceding the survey nor be under anti-malarial treatment.

Following enrollment, all households were geo-located using GPS (Garmin, Switzerland) instruments with up to 5 m of accuracy. Demographic information such as house type, age, and gender was collected from the head of the family. A short clinical assessment of each participant was conducted and information related to history of fever in the past two weeks and previous night, current febrile symptoms and previous night LLIN/bed net use were gathered. Apart from routine information, data on other malaria-related symptoms like headache, vomiting, and nausea were also gathered from the participants. Axillary temperature of all study participants was recorded with a digital thermometer (Dr. Diaz, Hemodiaz Life sciences Pvt. Ltd, India).

### Case definitions

Temperature of ≥99.5 °F/37.5 °C was considered a fever case. All RDTs and slide positives (presence of parasitaemia) with no febrile illness (during survey, previous night or past two weeks) were considered cases of asymptomatic malaria. All RDT or slide negatives who were positive by nested PCR with no febrile illness (during survey, previous night or past two weeks) were considered asymptomatic and sub-patent malaria cases [24, 25]. RDT positive with microscopy and PCR negative were considered as false positive.

### Blood sample collection

During the house visit, blood was collected for bivalent rapid diagnostic tests (RDT) (Bio-standard Diagnostic Pvt. Ltd, India) from each study participant, with a single finger prick according to the manufacturer’s instructions. Thick and thin blood smears were prepared for microscopic examination and stored in slide boxes. Two blood spots on Whatman qualitative filter paper no. 3 were collected from each participant and stored at 4 °C.

### Haemoglobin measurement

Haemoglobin (Hb) measurement was done with use of “Haemometer acc. to Sahli” (MARIENFELD, Laboratory glassware, Germany) during the household visit. 20 µl of blood was collected with the haemoglobin pipette, 0.1 N Hydrochloric acid (HCl) was added, mixed and compared with a haemoglobin comparator tube given by the manufacturer. Haemoglobin percentage less than 11 g/dl was categorized as anaemia and ≤9.5 g/dl was considered as moderate to severe anaemia [26].

### RDT-based diagnosis and treatment

RDTs were performed using a kit containing a monoclonal Anti *P. falciparum* histidine rich protein II (HRP-II) specific antibody and an anti *Plasmodium vivax* lactate dehydrogenase (p-LDH) specific antibody (Tulip group, Goa, India) to detect malaria infection.

All RDT/slide/PCR positive malaria cases were treated with anti-malarial according to national drug policy [*P. falciparum*: artesunate + sulfadoxine−pyrimethamine + primaquine; *P. vivax*: chloroquine + primaquine]. All the RDT positives were treated immediately at the point of collection and those detected positive by microscopy/PCR were treated immediately after the results were made available by the village health workers. Complete treatment of all positive cases was ensured by the village health workers and cross-checked by the supervisors.

### Microscopic examination

Geimsa-stained peripheral blood smears were examined under compound microscope (Olympus CX21 LED) at 1000× magnification to detect malaria parasites. Ring, trophozoite or gametocyte stages were identified and assessed for parasite density (parasites/µl blood) against 200 white blood cells (WBCs) considering the average of 8000 WBC/µl.

### Nested PCR assay

An area of 0.5 cm^2^ of three blood spots were punched and used for DNA isolation according to manufacturer instructions (HiPurA™ Forensic Sample Genomic DNA Purification Kit, Himedia, # MB524). DNA sample was eluted in 40 µl of DNase free water and stored at −20 °C for further use.

Nested-PCR was performed for identification of *P. falciparum* and *P. vivax* with the primers designed by Snounou et al. [27] and standard PCR conditions as suggested by Johnston et al. [17] with minor modifications. In the first-step of nested PCR, reaction mixture (15 µl) containing 1X Dream Taq master mixture (Thermo Scientific, # K1071), 0.2 µmol genus-specific primers rPLU5 and rPLU6 and 2 µl of isolated DNA from dried blood spot were mixed to amplify 1100 bp fragment. Then, from the product of the first-step PCR reaction, a 1:10 diluted aliquot was used as a template along with 0.2 µmol of species-specific primers rFAL1, rFAL2, rVIV1and rVIV2 for amplifying 205 bp for *P. falciparum* and 120 bp for *P. vivax* in nested-step PCR. In both the steps of nested PCR, following conditions were used: initial denaturation (95 °C/5 min), then 35 cycles of denaturation (95 °C/30 s), annealing (56 °C/30 s), extension (72 °C/60 s) and final extension (72 °C/10 min). The amplified DNA fragments were finally analysed by agarose gel (2%) electrophoresis stain.

### Statistical analysis

All data were entered in EpiData version 3.1 and transferred to SPSS version 20 (IBM, USA) for analysis. Mean and standard deviation (SD) of continuous variables and percentages of categorical variables were reported. Chi square (*X* ^*2*^) test or fisher’s exact test was performed to assess the difference in demographic characteristics in the study population. RDT, light microscopy and PCR results were compared. Diagnostic efficiency was calculated for all the three techniques. Univariate and multivariate logistic regression models were used to investigate the association between sub-patent malaria infection and exposure variables after adjusting for village. Odds ratios with 95% confidence intervals (CI) were reported.

### Ethical approval and source of funding

Funding was provided by the Bill and Melinda Gates Foundation (Grant Number-OPP 1062754). This study was undertaken as a part of a WHO-coordinated multi-country project and ethical clearance was obtained from the Institutional Ethics Committee of National Institute of Malaria Research (ICMR), New Delhi, India (ECR/NIMR/EC/2010/75).

## Results

### Study population demography

The present survey was conducted in the dry season when transmission intensity is assumed to be less, due to low vector density. From three adjacent villages, 437 individuals from 118 households were recruited in this study. Nearly 50% of the total population of 859 was covered in the survey. The majority of the individuals were 15 years and above (59.7%), followed by the 5–14 year group (27.4%). Out of 437 individuals, 57.1% were female, 48.2% reported previous night LLIN use which varied between villages. At the time of the survey, nearly two-thirds of the study population (67.9%) was reportedly anaemic and 20.5% (91) individuals reported fever last night (Table 3).

**Table 3:** Demographic and clinical characteristics of surveyed population (n = 437)

Variable	Category	n (%)
Demographic characteristics		
Total household covered		118
Age	1–4 years	57 (13)
	5–14 years	119 (27.3)
	15 years and above	261 (59.7)
Gender	Female	250 (57.2)
LLIN use	Yes	211 (48.3)
Moderate to severe anaemia (≤9.5 g/dl)	Yes	44 (10.1)
Reported previous night fever history	Yes	91 (20.5)
Clinical characteristics		
Total malaria positives (state method)		103/437 (23.6)
Total clinical malaria (with fever)		23/103 (22.3)
Total asymptomatic cases (without fever)		80/103 (77.7)
Total sub-patent malaria cases		45/103 (43.7)
Asymptomatic, sub-patent malaria cases		37/45 (82.2)
Symptomatic, sub-patent malaria cases(with fever)		08/45 (17.8)
Asymptomatic, sub-patent malaria cases among all positives		37/103 (35.9)
Symptomatic, sub-patent malaria cases (with fever) among all positives		08/103 (7.7)

### Prevalence of asymptomatic and sub-patent malaria in the study area

**Fig. 2:**
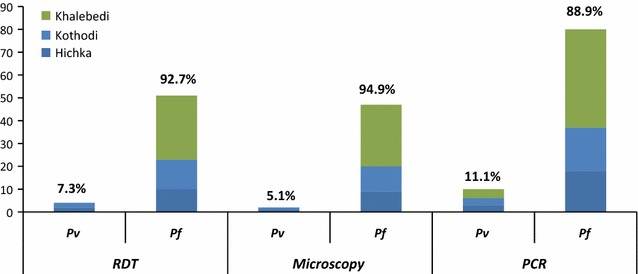
Distribution of *P. falciparum* and *P. vivax* positive cases by diagnostic techniques (n = 103)

55 persons were positive for malaria (either *P. falciparum* or *P. vivax)* by RDT. Among all febrile cases, one quarter (25.3%) was diagnosed positives for malaria. Among all 103 persons who were positive by PCR or microscopy, 43.7% (45/103 malaria positive cases) were classified as sub-patent malaria infections and more than 80% of sub-patent infections (37/45) were asymptomatic (Table 3). Figure 2 shows distribution of parasite (*P. vivax: P. falciparum*) by all three diagnostics, which suggests that the ration of *P. vivax* to *P. falciparum* was similar in RDT (7.3:92.7) and LM (5.1:94.9), but different with PCR (11.1: 88.9).

### Distribution of malaria and the diagnostic efficiency of three diagnostic methods

Table 4 shows distribution of malaria positive cases by diagnostic techniques. Nearly half of the malaria cases and sub-patent cases were contributed by one village Khalebedi (50.5). Sensitivity of RDT, microscopy and PCR are 53.3, 47.5 and 87.4% respectively. Measurement of agreement (Kappa) was higher between RDT and microscopy (66.7%, P < 0.001) as compared to RDT and PCR (40.2%, P < 0.001), and microscopy and PCR (43.6%, P < 0.001).

**Table 4:** Distribution of malaria and the diagnostic efficiency of three diagnostic methods (N = 437)

Variable	Category	Total positive^a^ n (%)	No. of cases with the following test results					
			RDT + Microscopy + PCR+	RDT + Microscopy − PCR+	RDT + Microscopy + PCR−	RDT − microscopy + PCR+	RDT − microscopy + PCR−	RDT – microscopy − PCR+
Positive cases	n	103 (23.6)	35	9	11	1	2	45
Presence of fever	Yes	23 (22.4)	5	5	4	1	0	8
	No	80 (77.6)	30	4	7	0	2	37

Diagnostic efficiency of three diagnostic methods			
Type of diagnostic methods	RDT	Microscopy	PCR
Diagnostic efficiency % (no of positives detected/total positives)	53.3% (55/103)	47.5% (49/103)	87.4% (91/103)

^a^RDT positive with microscopy and PCR negative were considered as false positive and not included as a malaria positive case

### Multivariate adjusted risk factor analysis of sub-patent malaria prevalence by village, age, moderate to severe anaemia, LLINs use, and *P. vivax* infection

Table 5 shows that age [OR (95% CI) 2.78 (1.25, 6.25); P = 0.012], moderate to severe anaemia status [4.33 (1.52, 12.35); P value 0.006], type of parasitic infection [3.96 (0.98, 15.93); P = 0.052] were risk factors for sub-patent infections identified by the univariate logistic regression analysis. Further, the multivariate adjusted analysis suggests that age 15 years [3.30 (1.33, 8.40); P = 0.010], moderate to severe anaemia [8.55 (2.34, 31.25); P = 0.001] and *P. vivax* infection [6.60(1.35, 32.26); P = 0.020] were independently associated with sub-patent malaria infections.

**Table 5:** Prevalence of sub-patent malaria infection by village, age, moderate to severe anaemia and LLINs use and type of parasite (n = 103)

Parameters	Category	Sub-patent malaria (%)	Patent malaria (N = 103)	Unadjusted odds ratio (95% CI)	P	Adjusted OR (95% CI)	P
Village	Hichka	13 (52.0)	25	1.2 (0.45–3.02)	0.756	0.91 (0.3–2.8)	0.863
	Kothodi	16 (61.5)	26	0.79 (0.30–2.1)	0.627	0.37 (0.1–1.35)	0.109
	Khalebedi	29 (55.8)	52	1			
Age (years)	≥15	26 (57.8)	45	2.78 (1.25–6.25)	*0.012*	3.30 (1.3–8.4)	*0.010*
	1–14	19 (32.8)	58	1			
Moderate to severe anaemia (≤9.5 g/dl)	Yes	15 (71.4)	21	4.33 (1.52–12.35)	*0.006*	8.5 (2.3–31.3)	*0.001*
	No	30 (36.6)	82	1			
LLINs use	No	29 (49.2)	59	1.69 (0.76–3.76)	0.197	1.01 (0.4–2.6)	0.985
	Yes	16 (36.4)	44	1			
Parasite type	*Pv*	8 (72.7)	11	3.96 (0.98–15.93)	*0.052*	6.60 (1.4–32.3)	*0.020*
	*Pf*	37 (40.2)	92	1			

Subject level-sub-patent malaria positives, *​P* value less than 0.05 are statistically significant

## Discussion

Keshkal sub-district is a comparatively low malaria endemic area with marked heterogeneity in reported malaria cases across the villages than adjacent sub-districts. Among four primary health centers (PHC’s) in Keshkal sub-district, Dhanora, where this study was undertaken, is the most malaria endemic area. State health department data showed that API varied from 0 to 16 per 1000 population in the past 5 years in the study villages. As part of the wider project in which this study is embedded, active fortnightly surveillance for malaria was carried out since 2015. During the routine surveillance, only one or two febrile malaria positives were detected in the study area every month, but in a cross sectional survey conducted in December 2015 the overall positivity rate ranged from 8.6 to 45.6% in the study sites, which suggests the presence of asymptomatic and sub-patent infections in the community. Hence, the present study was undertaken to estimate the proportion of sub-patent infections, showing that the burden of asymptomatic and sub-patent infections was high compared to what was reported in routine surveillance of fever cases. This could partially be attributed to negligence in health seeking behavior and distance to nearest health facility from the village.

A significant percentage of those infected (77.6%) were harbouring *Plasmodium* parasites without any signs or symptoms of malaria. These inhabitants do not visit the diagnostic facility or seek any anti-malarial treatment due to absence of any symptoms. These individuals constitute a reservoir of parasites and hence a source of infection for transmission. The chances of getting the infection would be higher if they share a common household unit. If a household has a member infected with malaria, it is more likely that there will be other infected persons clustered in that unit [11, 12, 28].

The diagnostic efficiency of light microscopy and RDT is compromised in areas where a substantial number of asymptomatic and sub-patent cases exist [29]. This study has demonstrated that the diagnostic efficiency of RDT and microscopy is only 53.3 and 47.5%, respectively. Hence, PCR could overcome this problem due to its high diagnostic efficiency to detect sub-patent parasitaemia in the blood. In the general protocol for blood examination of malaria parasites, the volume of blood during microscopy peripheral blood smear examination and DNA extraction for PCR are 0.12–0.25 and 5–100 µl respectively, and the parasite detection limit is higher for PCR (0.01–0.2 parasite per µl) compared with microscopy is approximately 4–10 parasite per µl) [15, 30].

Parasite detection by microscopy is limited for a number of reasons, such as its inability to detect low density of parasite infected red blood cells, and the loss of a number of parasite materials during slide staining especially in presence of scanty parasitaemia [31]. On the other hand the general use of the more sensitive diagnostics of PCR is precluded by its high cost and the need for local access to appropriate laboratories. This study has found that 13 out of 103 cases were possibly missed by PCR which were diagnosed positive by RDT, this could be due to circulating HRP2 after parasite clearance [32]. Sensitivity of PCR technique also depends on the amount of parasites in the volume of blood used for DNA extraction and the concentration of extracted parasite DNA [13]. The newly developed highly sensitive RDT or loop-mediated isothermal amplification (LAMP), once it is available for the field purposes, could be an option to improve the diagnosis of sub-patent infections in hot spots where the occurrence and presence of submicroscopic malaria is common [32].

In areas with asymptomatic and sub-patent infections, mass drug administration could be a viable alternative to active case detection, since ACT uses an effective drug combination for clearance of sub-patent gametocytes in these reservoirs [33, 34]. Secondly, reactive case detection (RACD), is a more targeted form of active case detection and could, therefore, be used along with highly sensitive RDT to try to identify those cases that would otherwise be missed [35, 36].

Risk factor analysis shows that older age (15 years and above) and moderate to severe anaemia (≤9.5 g/dl) and *P. vivax* rather than *P. falciparum* are important risk factors of sub-patent infections. Though *P. falciparum* infection is more common in the area, *P. vivax* infection was associated with sub-patent infection. This could be due to this species’ capability to develop gametocytes at very early stage of erythrocytic schizogony, before the onset of any symptoms. Similar observations were reported in a study in Central Vietnam [37]. Sub-patent infections were also associated with adolescents and adults as opposed to younger children. This is possibly because age is a proxy for acquired clinical immunity due to repeated infections and, therefore, resulting in partial parasite clearance in these individuals [13].

Asymptomatic malaria can perpetuate unstable transmission conditions and thus frustrate elimination efforts unless these reservoirs are targeted. This study has added to the evidence that in areas of low transmission malaria surveillance programmes need to be strengthened to identify hotspots, diagnose asymptomatic cases and investigate foci of transmission.

## Limitations

This study was conducted with small sample size in the selected tribal villages where the annual parasite incidence varied from 0 to 16 per 1000 population; hence generalization of study findings in the wider population should be done with adequate caution. Nonetheless, results are relevant in Indian context which has decided for malaria elimination in near future.

## Conclusions

A substantial proportion of asymptomatic and sub-patent malaria carriers are present in the population living in the study area with low transmission. The burden of hidden parasite reservoirs is a major challenge for malaria elimination programmes in the country. Hence strategies for locating hotspots of asymptomatic malaria to control and eliminate such foci of reservoirs should be a focus of active case management in forested rural tribal areas.

## Abbreviations

PCR: polymerase chain reaction
LLINs: long-lasting insecticidal nets
IRS: indoor residual spraying
ACT: artemisinin-based combined therapy
RDT: rapid diagnostic test
LM: light microscopy
PHC: Primary Health Centres
WBC: white blood cells
WHO: World Health Organization
API: annual parasitic incidences
SPR: slide positivity rate
BSC: blood slide collection
TP: total positive cases
